# Supplementary material for: Prevalence, locations and predictors of attitudes accepting both intimate partner violence and additional forms of violence against women and girls in South Sudan: a geospatial analysis
Source: PLOS Glob Public Health. 2025 Apr 9;5(4):e0004144. doi: 10.1371/journal.pgph.0004144 (PMC11981127; doi:10.1371/journal.pgph.0004144)
Supplement: S1 Table — Description: For each state of the number of supervision areas (SAs), number of interviews per SA, total sample size, and maximum 95% Confidence Interval. (DOCX) [file pgph.0004144.s001.docx]

S1Table: Sample size estimation

| **State** | **Nº SA** | **Nº interviews to collect per SA** | **People to survey** | **95%CI does not exceed** |
| --- | --- | --- | --- | --- |
| Central Equatoria | 6 | 19 | 114 (6x19) | +9.2% |
| Western Equatoria | 10 | 19 | 190 (10x19) | +7.1% |
| Eastern Equatoria | 8 | 19 | 152 (8x19) | +7.9% |
| Jonglei | 9 | 19 | 171(9x19) | +7.9% |
| Lakes | 8 | 19 | 152 (9x19) | +7.9% |
| Northern Bar el-Ghazal | 5 | 19 | 95 (5x19) | +10.1% |
| Western Bar el-Ghazal | 5 | 19 | 95 (5x19) | +10.1% |
| Unity | 7 | 19 | 133 (7x19) | +8.5% |
| Upper Nile | 13 | 19 | 247 (13x19) | +6.2% |
| Warrap | 6 | 19 | 114 (6x19) | +9.2% |
| Abyei AA | 4 | 24 | 96 (4x24) | +10% |
| Greater Pibor AA | 4 | 24 | 96 (4x24) | +10% |
| Ruweng AA | 4 | 24 | 96 (4x24) | +10% |
| Total | 89 | - | 1751 | - |

AA= Administrative area

SA= Supervision area
