# Supplementary material for: Prevalence, locations and predictors of attitudes accepting both intimate partner violence and additional forms of violence against women and girls in South Sudan: a geospatial analysis
Source: PLOS Glob Public Health. 2025 Apr 9;5(4):e0004144. doi: 10.1371/journal.pgph.0004144 (PMC11981127; doi:10.1371/journal.pgph.0004144)
Supplement: S1 Text — Description: Sensitivity analysis of the prevalence, spatial analysis results and predictors of attitudes accepting both intimate partner violence and additional forms of violence against women and girls in South Sudan. (DOCX) [file pgph.0004144.s002.docx]

**S1 TEXT**

**Sensitivity Analysis of the Prevalence, Spatial Analysis and Predictors of the Overlap Between Attitudes Accepting Intimate Partner Violence and at Least One Additional Expression of Violence Against Women and Girls in South Sudan**

**Fig A** Number of people included **……………………………………………………………………………..…3**

**Fig B** Sensitivity analysis with SatScan**…………………………………………………………………………..6**

**Table A** Sensitivity analysis of the prevalence**…..……………………………………….………………….4**

**Table B** Sensitivity analysis of estimation of Global Moran`s I **……………………..……………….4**

**Table C** Sensitivity analysis of estimation of Getis and Ord`s local G***…………………………….5**

**Table D** Main analysis results of the multilevel logistic regression**……………………………..….7**

**Table E** First sensitivity analysis results of the multilevel logistic regression**…………………..8**

**Table F** Second sensitivity analysis results of the multilevel logistic regression**………………9**

We conducted a sensitivity analysis in order to assess the robustness of our findings and determine if the method used to deal with missing data impacted the results.

**METHODS**

We used two different approaches to compare with the results displayed in the study`s primary analyses. These were conducted on the outcome “overlap of attitudes accepting intimate partner violence (IPV) and at least one other expression of violence against women and girls (VAWG)” in each step of the analysis plan:

1. Estimate the prevalence.
2. Estimate Global Moran`s I and Getis and Ord`s Local Gi*.
3. Identify clusters using SatScan.
4. Run a multilevel logistic regression.

In the first approach to our sensitivity analysis, we excluded observations with any missing data (i.e., answers with “I do not” to any question about justifying a husband hitting or beating his wife, as well as justifying child marriage, raiding women for women during cattle rustling, or female genital mutilation) which kept 76.7% from the main data (n=2654).

In the second approach, we used the same database used in the study`s primary analyses in which a state’s median predominant answer was the missing value, but we excluded Maban, Tonj South, and Yei counties since most of their data was a missing value. This approach retained 96.7% of the data (n= 3348).

**RESULTS**

Fig A displays: A) the numbers of people surveyed (where responses “I do not” were imputed using the state’s median value of the predominant answer for the missing (or do not know) responses), which will be presented as “main analysis” in the following steps; B) the number of people excluded in the “first sensitivity analysis”; and C) the number of people excluded in the “second sensitivity analysis”.


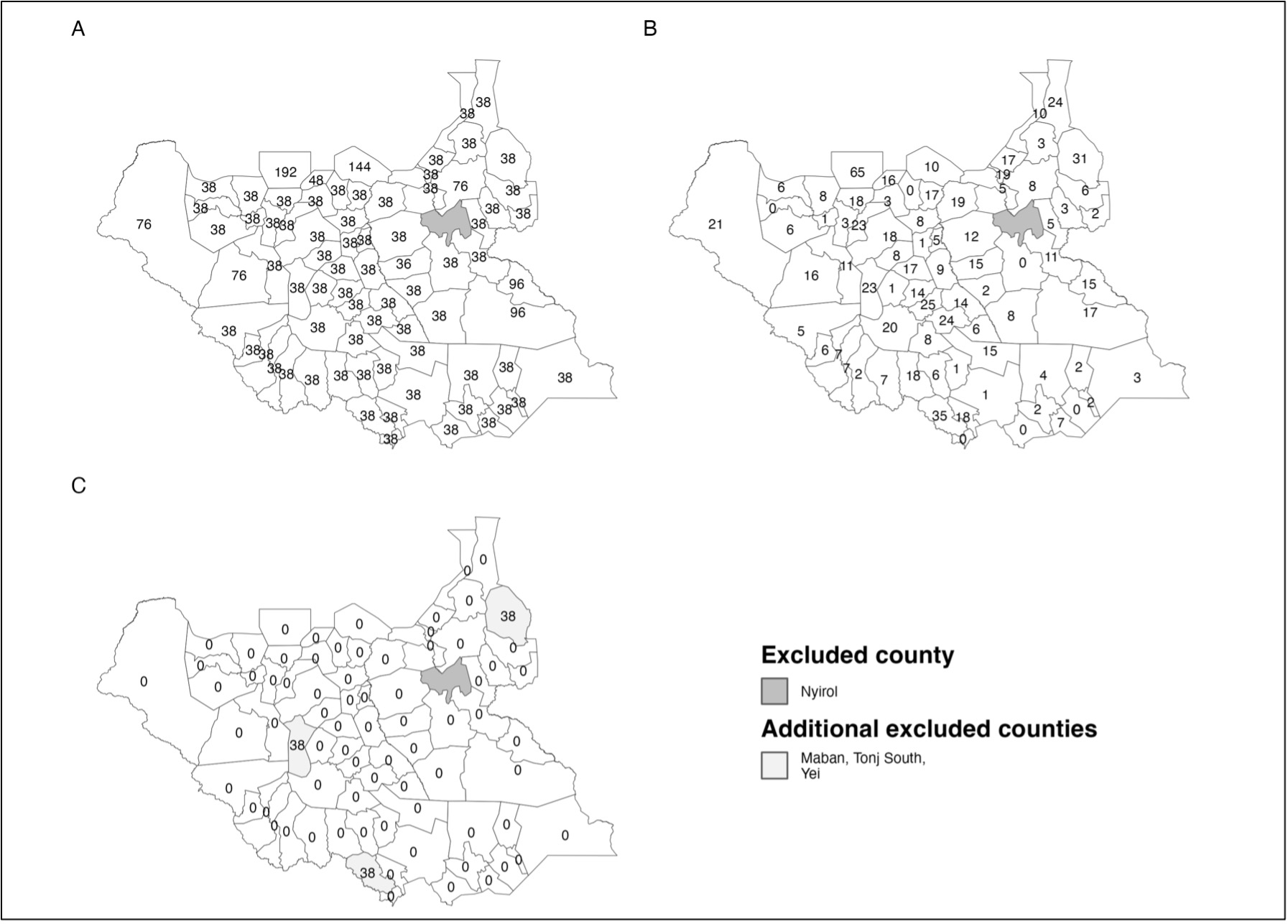


***Fig A*** *. Number of people included:* ***A****) number of people included in the main analysis,* ***B)*** *Number of people excluded from the first sensitivity analysis,* ***C)*** *Number of people excluded from the second sensitivity analysis*

1. **Sensitivity analysis of the prevalence of overlapping between attitudes accepting IPV and attitudes accepting at least one other expression of VAWG**

The estimated prevalence of overlapping between attitudes accepting IPV with at least one other expression of VAWG in the first and secondary sensitivity analyses were 1.26% and 0.56% higher than in the main analysis, respectively (Table A).

***Table A.*** *Sensitivity analysis of the prevalence of overlapping between attitudes accepting IPV and attitudes accepting at least one other expression of VAWG.*

|  | Main Analysis  N=3462 | First Sensitivity analysis  N= 2654 | Second Sensitivity analysis  N= 3348 |
| --- | --- | --- | --- |
| Prevalence (%) | 34.72 | 35.98 | 35.28 |
| 95%CI | 33.13-36.30 | 34.15-37.81 | 33.65-36.89 |

1. **Sensitivity analysis of estimation of Global Moran`s I of overlapping between attitudes accepting IPV and attitudes accepting at least one other expression of VAWG**

Table B shows the Global Moran’s I output for the three analyses. The Moran’s Index uses a scale ranging from 0 to 1 with 1 indicating a high level of clustering. Our analyses did not detect important differences among the three analysis approaches.

***Table B.*** *Sensitivity analysis of estimation of Global Moran`s I of overlapping between attitudes accepting IPV and attitudes accepting at least one other expression of VAWG.*

|  | Main Analysis | First Sensitivity analysis | Second Sensitivity analysis |
| --- | --- | --- | --- |
| Moran`s Index | 0.23 | 0.21 | 0.23 |
| z-score | 3.38 | 3.06 | 3.19 |
| p-value | <0.001 | 0.002 | 0.001 |

1. **Sensitivity analysis of estimation of Getis and Ord`s Local G* of overlapping between attitudes accepting IPV and attitudes accepting at least one other expression of VAWG**

Our results in the first sensitivity analysis differed slightly from the main analysis since the counties Kapoeta East, Mayendit, and Leer became hotspots with 95% confidence (rather than 90% confidence). Kapoeta South was kept as a hotspot with 90%, and Juba as a coldspot with 90%. By contrast, Kapoeta North lost the category of hotspot. The results of the second sensitivity analysis were the same as in the main ones (see Table C).

***Table C.*** *Sensitivity analysis of estimation of Getis and Ord`s local G* of overlapping between attitudes accepting IPV and attitudes accepting at least one other expression of VAWG.*

|  | Main analysis | First sensitivity analysis | Second sensitivity analysis |
| --- | --- | --- | --- |
| Hotspots with 95% confidence | - | Kapoeta East, Mayendit and Leer | - |
| Hotspots with 90% confidence | Kapoeta East, Kapoeta North, Kapoeta South, Mayendit and Leer | Kapoeta South | Kapoeta East, Kapoeta North, Kapoeta South, Mayendit and Leer |
| Coldspots with 90% confidence | Juba | Juba | Juba |

1. **Sensitivity analysis of the SatScan analysis of** **overlapping between attitudes accepting IPV and attitudes accepting at least one other expression of VAWG**

In our main results (Fig B.A), SatScan analysis displayed 6 clusters with p-value <0.05 of the outcome “overlap between attitudes accepting IPV and attitudes accepting at least one other expression of VAWG”. Two of them have Log likelihood ratio (LLR) >100, 1 between 40-100 LLR, and 3 <40 LLR (Fig B.B). LLR allow identifying areas with the most likely to be a cluster, the highest LLR suggest the most likely to be a cluster. ^(1)^

The primary clusters include Kapoeta East, Kapoeta South, Kapoeta North, Budi, Pibor, and Ikotos counties. These clusters were in the coordinates 5.138396 N, 34.652377 E, with a radius of 208.80 km. People living in these clusters have 2.71 higher risk of having attitudes accepting overlapping VAWG than those living outside of them.

Fig B.B shows the 7 clusters found in the first sensitivity analysis with p-value <0.05. Six of 7 clusters were in the same locations as the main analysis. The additional clusters were located in the area corresponding to Pariang and Guit counties. Two of them have LLR>100, and 5 have LLR< 40. The primary clusters include the same counties as in our main results. People living in these clusters have a 2.62 higher risk of having attitudes accepting overlapping VAWG than those living outside of them.

The second sensitivity analysis (Fig B.C) showed no differences from the main analysis, as the same six clusters that were statically significant were found and had the same LLR range. Additionally, the primary clusters were identified in the same location, and the people living in these clusters have a 2.67 higher risk of having attitudes accepting overlapping VAWG than those living outside of them.


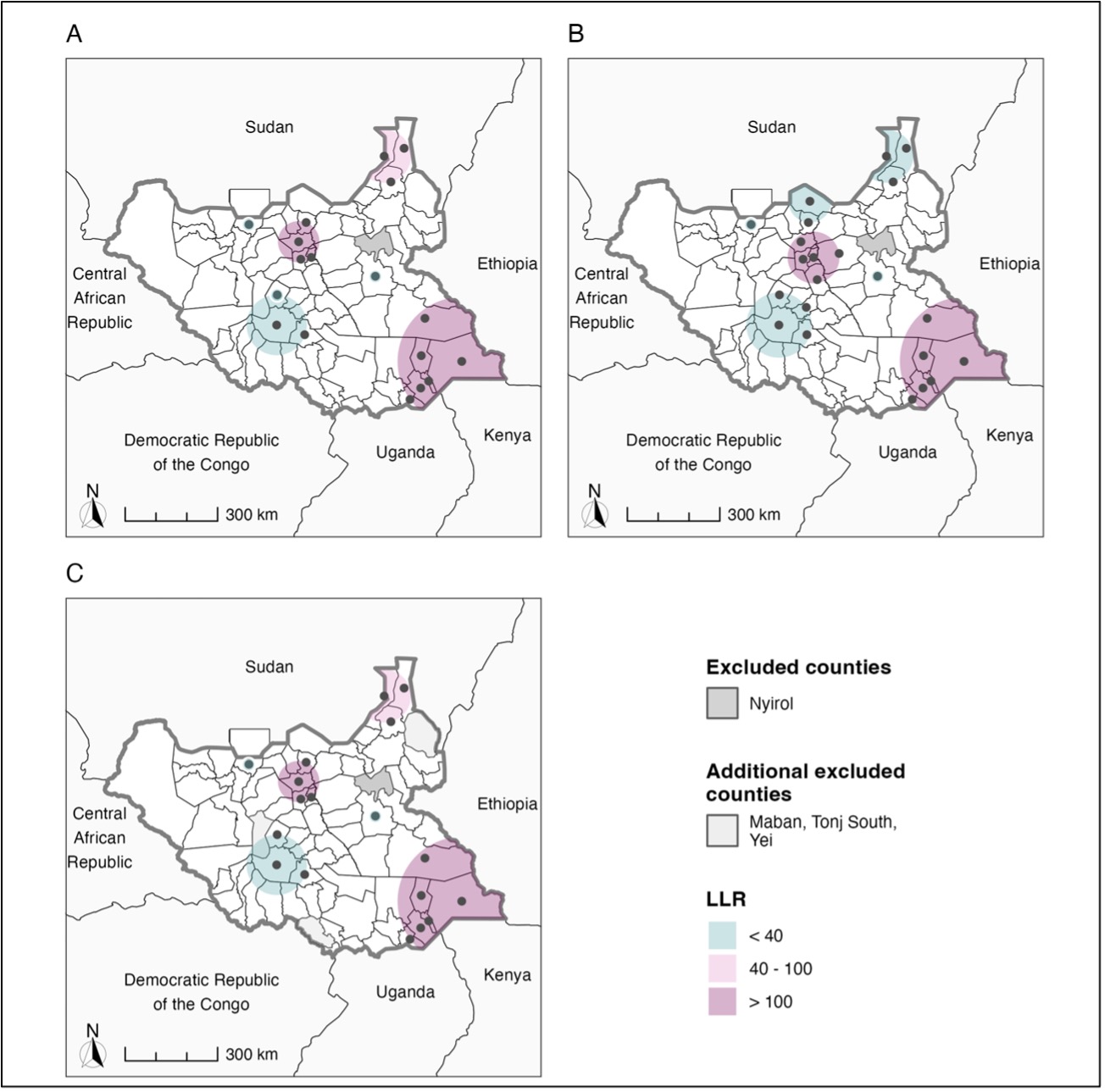


***Fig B.*** *Sensitivity analysis with SatScan****. A)*** *Main analysis,* ***B)*** *First sensitivity analysis,* ***C)*** *Second sensitivity analysis.*

1. **Sensitivity analysis of the multilevel logistic regression of** **overlapping between attitudes accepting IPV and attitudes** **accepting at least one other expression of VAWG**

Model III (both individual and community-regional level data) had the highest log-likelihood, which means it was the best fit for data ^(2)^ (see Table D). People married, cohabited, or living together as well as people who were widowed, divorced, or separated displayed a higher odds of overlapping between attitudes accepting IPV and attitudes accepting at least one other expression of VAWG (aOR=1.40; 95%CI=1.03-1.90 and aOR=1.76; 95%CI:1.05-2.93, respectively) compared to the reference group of people who never married. Also, people who live in counties where the largest portion of the community has any level of education (non-illiteracy) had lower odds (OR 0.26, 95%CI: 0.09-0.70) of overlapping between attitudes accepting IPV and attitudes accepting at least one other expression of VAWG compared to people who live in counties where illiteracy was predominate.

***Table D****. Main analysis results of the multilevel logistic regression (aOR, 95%CI).*

| **Characteristics**  (n=3462) | **Model 0**  **aOR**  **(95%CI)** | **Model I**  **aOR**  **(95%CI)** | **Model II**  **aOR**  **(95%CI)** | **Model III**  **aOR**  **(95%CI)** |
| --- | --- | --- | --- | --- |
| Age |  | 0.99  (0.98-1.00) |  | 0.99  (0.98-1.00) |
| Gender  Men  Women |  | Ref  1.03  (0.84-1.26) |  | Ref  1.03  (0.84-1.27) |
| Marital Status  Never married  Married, cohabited or living together  Widowed, divorced or separated |  | Ref  **1.40***  **(1.04-1.90)**  **1.75***  **(1.05-2.93)** |  | Ref  **1.40***  **(1.03-1.90)**  **1.76***  **(1.05-2.93)** |
| Education level  Illiteracy  Primary  Secondary or upper |  | Ref  0.87  (0.67-1.13)  0.82  (0.60-1.12) |  | Ref  0.90  (0.70-1.16)  0.84  (0.62-1.14) |
| Community literacy level  Illiteracy  Non-Illiteracy |  |  | Ref  **0.24***  **(0.09-0.63)** | Ref  **0.26***  **(0.09-0.69)** |
| Region  The Greater Equatoria  The Greater Upper Nile  The Greater Bahr El-Ghazal |  |  | Ref  2.39  (0.75-7.63)  0.88  (0.25-3.09) | Ref  2.41  (0.75-7.72)  0.91  (0.26-3.20) |
| Random effects results  County Variance (95%CI)  ICC | 5.12  (3.47-7.60)  0.61 | 5.04  (3.41-7.45)  0.61 | 4.23  (2.85-6.27)  0.56 | 4.23  (2.85-6.26)  0.56 |
| Model fitness  Log-likelihood | -1551.3834 | -1545.7192 | -1545.1118 | -1539.8757 |

* p-value < 0.05

Overall, in the first sensitivity analysis (Table E), the results display some similarities with the main ones. Model III was also the best fitting model. Yet, the factors involved “married, cohabited or living together” lost the significance (OR 1.20, 95%CI: 0.84-1.73). Widowed, divorced, or separated continued as a risk factor (OR=2.06, 95%CI: 1.13-3.77). Community literacy level also lost significance (OR=0.34, 95%CI: 0.12-1.00).

***Table E****. First sensitivity analysis results of the multilevel logistic regression (aOR, 95%CI).*

| **Characteristics**  (n=2654) | **Model 0**  **aOR**  **(95%CI)** | **Model I**  **aOR**  **(95% CI)** | **Model II**  **aOR**  **(95% CI)** | **Model III**  **aOR**  **(95% CI)** |
| --- | --- | --- | --- | --- |
| Age |  | 0.99  (0.97-1.00) |  | 0.99  (0.97-1.00) |
| Gender  Men  Women |  | Ref  1.04  (0.81-1.33) |  | Ref  1.05  (0.82-1.34) |
| Marital Status  Never married  Married, cohabited or living together  Widowed, divorced or separated |  | Ref  1.21  (0.84-1.75)  **2.06***  **(1.13-3.77)** |  | Ref  1.20  (0.84-1.73)  **2.06***  **(1.13-3.77)** |
| Education level  Illiteracy  Primary    Secondary or upper |  | Ref  0.80  (0.59-1.08)  0.76  (0.23-1.01) |  | Ref  0.82  (0.61-1.11)  0.78  (0.55-1.12) |
| Community literacy level  Illiteracy  Non-Illiteracy |  |  | Ref  **0.31***  **(0.11-0.92)** | Ref  0.34  (0.12-1.00) |
| Region  The Greater Equatoria  The Greater Upper Nile  The Greater Bahr El-Ghazal |  |  | Ref  2.52  (0.70-9.10)  1.10  (0.29-4.25) | Ref  2.54  (0.70-9.22)  1.12  (0.29-4.32) |
| Random effects results  County Variance (95%CI)    ICC | 6.14  (4.05-9.30)  65.10 | 6.03  (3.98-9.16)  64.73 | 5.33  (3.51-8.12)  61.87 | 5.32  (3.50-8.09)  61.79 |
| Model fitness  Log-likelihood | -1161.01 | -1154.4804 | -1157.0546 | -1150.824 |

* p-value < 0.05

In the second sensitivity analysis (Table F), Model III was the best fitting model. While the association with marital status married, cohabited, or living together was not significant (OR=1.34, 95%CI: 0.99-1.83), the association with widowed, divorced or separated was significant (OR=1.80, 95%CI: 1.07-3.02). Likewise, the community literacy also kept significance (OR=0.25, 95%CI: 0.09-0.69).

***Table F.*** *Second sensitivity analysis results of the multilevel logistic regression (aOR, 95%CI).*

| **Characteristics**  (n=3348) | **Model 0**  **aOR**  **(95%CI)** | **Model I**  **aOR**  **(95% CI)** | **Model II**  **aOR**  **(95% CI)** | **Model III**  **aOR**  **(95% CI)** |
| --- | --- | --- | --- | --- |
| Age |  | 0.99  (0.98-1.00) |  | 0.99  (0.98-1.00) |
| Gender  Men  Women |  | Ref  1.06  (0.86-1.31) |  | Ref  1.07  (0.87-1.31) |
| Marital Status  Never married  Married, cohabited or living together  Widowed, divorced or separated |  | Ref  1.35  (0.99-1.84)  **1.79***  **(1.06-3.01)** |  | Ref  1.34  (0.99-1.83)  **1.80***  **(1.07-3.02)** |
| Education level  Illiteracy  Primary    Secondary or upper |  | Ref  0.87  (0.67-1.13)  0.82  (0.60-1.13) |  | Ref  0.89  (0.69-1.16)  0.84  (0.62-1.16) |
| Community literacy level  Illiteracy  Non-Illiteracy |  |  | Ref  **0.23***  **(0.08-0.64)** | Ref  **0.25***  **(0.09-0.69)** |
| Region  The Greater Equatoria  The Greater Upper Nile  The Greater Bahr El-Ghazal |  |  | Ref  2.58  (0.78-8.60)  0.92  (0.25-3.42) | Ref  2.62  (0.79-8.71)  0.95  (0.26-3.53) |
| Random effects results  County Variance (95%CI)  ICC | 5.37  (3.60-8.00  62.02 | 5.29  (3.55-7.90)  61.69 | 4.44  (2.97-6.64)  57.46 | 4.44  (2.97-6.64)  57.45 |
| Model fitness  Log-likelihood | -1494.245 | -1488.578 | -1488.228 | -1482.915 |

* p-value < 0.05

**DISCUSSION AND CONCLUSION**

The first and second sensitivity analyses did not reveal important differences with our main results when estimating prevalence and Global Moran’s I. Little more information was gained by using any of the alternative approaches for dealing with missing data.

Using Getis and Ord`s local G*, the first sensitivity analysis showed only slight differences from our initial results. The same counties are classified as hotspots (except for Kapoeta North). Yet, the confidence level of the hotspots was higher (95% confidence vs 90% in the initial results). The second sensitivity analysis showed the same results as our main results.

Using SatScan, the first sensitivity analysis was slightly different from the main ones. There are 7 clusters rather than 6 (as in the main results). In addition, p-values are significant but with different levels of LLR. The second sensitivity analysis showed the same results as our initial results.

Model III had the best fit using in the multilevel logistic regression. However, there were differences among the explanatory variables. In the first sensitivity analysis, only “widowed, divorced, or separated” was associated with the overlap between attitudes accepting IPV and attitudes accepting other expression of VAWG. By contrast, in the second sensitivity approach, “widowed, divorced, or separated” and “community literacy level” revealed a significant association. However, in our main results, we estimated the already mentioned exposures; in addition, “married, cohabited or living together”, was also statistically significant.

In summary, the approach for dealing with missing values as reported in the main manuscript is acceptable. Especially compared to the second sensitivity analysis. The slight differences displayed with the first one could result from the higher observations eliminated (808 observations (23.3%) were eliminated in the first sensitivity analysis v/s 114 (3.3%) in the second one)

In this case, conducting an analysis using complete case analysis, mainly when the missing mechanism is not completely at random, has the potential to introduce bias and result in a loss of efficiency in parameter estimates (reduce sample size). ^(3)^

**REFERENCES**

1. Kulldorff M. A spatial scan statistic. Commun Stat Theory Methods. 1997;26(6):1481-96.

2. Goldstein H. Multilevel Statistical Models, 2010.

3. Mukaka M, White SA, Terlouw DJ, Mwapasa V, Kalilani-Phiri L, Faragher EB. Is using multiple imputation better than complete case analysis for estimating a prevalence (risk) difference in randomized controlled trials when binary outcome observations are missing? Trials. 2016;17(1):341.
