# Supplementary figures and images for: Prevalence, locations and predictors of attitudes accepting both intimate partner violence and additional forms of violence against women and girls in South Sudan: a geospatial analysis
Source: PLOS Glob Public Health. 2025 Apr 9;5(4):e0004144. doi: 10.1371/journal.pgph.0004144 (PMC11981127; doi:10.1371/journal.pgph.0004144)

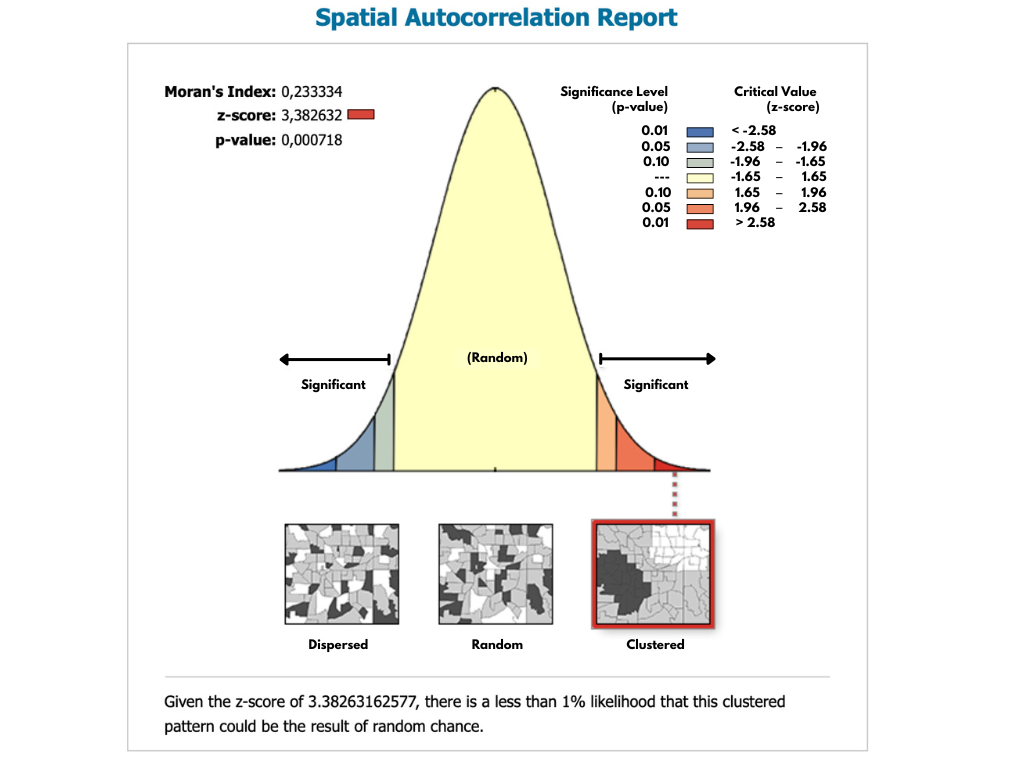

Supplement: S1 Fig — (TIF) [file pgph.0004144.s003.tif]
