# Supplementary material for: Prevalence, locations and predictors of attitudes accepting both intimate partner violence and additional forms of violence against women and girls in South Sudan: a geospatial analysis
Source: PLOS Glob Public Health. 2025 Apr 9;5(4):e0004144. doi: 10.1371/journal.pgph.0004144 (PMC11981127; doi:10.1371/journal.pgph.0004144)
Supplement: Attachment — Submitted filename: Prevalence locations and predictors of attitudes comments.pdf [file pgph.0004144.s004.pdf]

Manuscript PGPH-D-24-01742,

Title. 'Prevalence, locations and predictors of attitudes accepting both intimate partner violence and additional forms of violence against women and girls in South Sudan: a geospatial analysis

Review comments

ABSTRACT

1. Page 3, line 23-51: Add the sub titles of the abstract such as Introduction, Objective, Methodology, Result, Conclusion and recommendation…
2. Page 2, line 24-28: Rewrite the sentence… "if attitudes accepting IPV overlap with attitudes accepting additional forms of VAWG …" like "This research aimed to determine the prevalence of attitudes accepting IPV overlap with additional forms of VAWG…"
3. Page 2, line 37-38: Rewrite the sentence… The prevalence of attitudes accepting IPV overlapped with attitudes accepting at least one other form of VAWG was 34.72% (95%CI: 33.14-36.34) ~~of the time.~~
4. Page 2, line 41-44: What do you mean OR? Is it Adusted odds ratio or Crude odds ration? Make it clear for the readers. It can be corrected as "People married, cohabiting or living together (AOR(~~OR~~=1.40, 95%CI=1.04-1.90) as well as people widowed, divorced or separated (AOR ~~(OR~~= 1.75, 95%CI=1.05-2.93) were associated with attitudinal overlaps. Conversely, communities with any formal education were associated with a lower odds of overlapping (AOR~~ (OR~~=0.26. 95%CI=0.09-0.70)."
5. Page 3, line 48-50: Please write your conclusions and recommendations based on your findings.

Introduction

6. Page 4, line 61-66: Why you use all paragraphs as capital letters? Please write your basic reasons unless use the small letters as you used in other paragraphs.
7. Page 4, line 63-65: Please add other risk factors identified after reviewing different recent literatures.

MATERIALS AND METHODS

Study design and sample size

8. Page 6, line 131-132: Of the interviewed participants, how many of them where females and males individually?

9. Page 7, line 136-146: The sampling technique is not clearly explained. The way of the determining the sampling size was not also clearly explained. So, please rewrite in detail for understanding for the readers more in figures.
10. Page 7, line 156: How do you check the consistency of data and languages translations? Explain more within the manuscript.
11. Page 10, line 208: The prevalence of what? Please rewrite the word aligned with your objectives.
12. Page 10, line 215: The Spatial analysis of what? Please rewrite the word aligned with your objectives.
13. Page 11, line 237: The Multilevel regression of what? Please rewrite the word aligned with your objectives.
14. Page 12, line 251-252: When would be the multicollinearity test conducted? Is it during Multilevel regression or before? Write your explanation and corrections in brief.

RESULTS

15. Page 12, line 270: Which sentence is more appropriate? Characteristics of the sample or Characteristics of the participants? Explain your justification.
16. Page 13, line 271-272: What is your response rate?
17. Page 13, line 278-281: Table 1 is not written as standard of writing tables. Please rewrite the table.
18. Page 14, line 282: The prevalence of what? Please rewrite the word with the topic you want to explain.
19. Page 13, line 290-291: update the table with standard way of table presentation (the comment is for all tables).
20. Page 16, line 308-326: There is the figure 1, 2 and 3? Please add the figures within your manuscript.
21. Page 18, line 341-342: Please clarify OR whether Adjusted or crude odds ratio.
22. Page 18-20, line 346-349: Please add the p-value of individual independent variables (Both Bilevel and Multilevel logistic regression) with in the table for clear understanding of the association for general readers.
23. Page 21, line 359-363: Please clarify OR whether Adjusted or crude odds ratio? The comment is applicable throughout the manuscript.

24. Page 22, line 397-401: I am not clear about your explanation here. It that result or discussion component of the manuscript? Please discuss your finding in detail as compare to other research findings both for higher and level prevalence of overlapping acceptance of IPV and at least one other form of VAWG and lastly suggest your reasons for it.
25. Page 22, line 399: Only use one style of reference either Vancouver or Harvard style. Don't use both within single research.
26. Page 24, line 426-432: Show the findings in table or add the table number which shows this finding for general readers.
27. Page 25, line 467: Use the most recent world bank report if available.
28. Page 25, line 474-478: Make sure that you discussed are align with your findings. Don't add or explain any other factors which are not related with your findings.
29. Page 26, line 493-498: This is not a strength of the study rather it is the recommendation. Please use this at a recommendation part of your research. Note that, your recommendation should be based on your findings.
30. Page 28, line 527-228: please add the title of recommendation here or together as conclusion like "Conclusions and recommendations" on line 510.

As general comments, some components of the manuscripts are messing such as

1. Ethical considerations
2. Abbreviations/Acronyms
3. Availability of data and materials
4. Funding
5. Computing interest
6. Authors contributions
7. Authors information if needed
