# Supplementary material for: Prevalence, locations and predictors of attitudes accepting both intimate partner violence and additional forms of violence against women and girls in South Sudan: a geospatial analysis
Source: PLOS Glob Public Health. 2025 Apr 9;5(4):e0004144. doi: 10.1371/journal.pgph.0004144 (PMC11981127; doi:10.1371/journal.pgph.0004144)
Supplement: Attachment — Submitted filename: Peer_Review_PGPH-D-24-01742.pdf [file pgph.0004144.s005.pdf]

Peer Review for "Understanding attitudes accepting both IPV and VAWG" [PGPH-D-24-01742]

Manuscript Title: *Prevalence, locations and predictors of attitudes accepting both intimate partner violence and additional forms of violence against women and girls in South Sudan: a geospatial analysis*

1. General Overview

In this original research article, the authors present novel analyses of IPV and VAWG in South Sudan. Using the National Household Survey South Sudan 2020, and a suite of spatial analyses the authors conclude that acceptance of IPV and at least one other form of VAGW is spatially clustered. The authors provide foundational policy recommendations and insights that can inform women's empowerment and support international policy initiatives.

2. Introduction

The authors properly situate Violence Against Women and Girls as a significant global public health issue and human rights violation. Causal investigations of VAGW focus on gender dynamics and patriarchal normativity. Considering these issues, the authors indicate three aims: (i) calculate attitudinal prevalence of acceptance of IPW and overlapping attitudes toward IPW and one form of VAGW, (ii) analyze clusters, and (iii) identify relevant explanatory factors for these attitudes.

3. Methods

Study Design

This study is a cross-sectional study on a secondary analysis of data from the National Household Survey of South Sudan, 2020. This dataset is appropriate for the research aims and goals. Outcomes are clearly defined. Geographical groupings are logical and supported by literature.

Sampling and Data Collection

LQAS is *somewhat insufficiently* explained as a sampling method. A brief passage on its justification would support the readership's comprehension. For example, "It is commonly applied in public health and manufacturing to assess whether certain quality or performance thresholds are met without requiring a full population survey."

The use of PPS is appropriate for this analysis. Importantly, PPS requires accurate population estimates to determine sampling probabilities. If the population data is outdated, incomplete, or inaccurate (authors indicated using *2008 census* for a conflict-affected country like South Sudan), the sample may not represent the current population distribution. In regions with high migration, displacement, or population changes, PPS might oversample or under sample certain areas. PPS tends to under sample sparsely populated areas, which may lead to different results. Despite this, these methodological concerns should be discussed more completely.

Spatial Analysis

The use Local Indicators of Spatial Association (LISAs) is a appropriate class of spatial analysis for addressing the aims of the present study. Global Moran's I is an appropriate measure of clustering. Getis and Ord's local Gi* are appropriate measures for Hotspot Analysis. No issues are noted here.

Multilevel Analysis

Multilevel models are appropriately described and statistically sound. The authors indicate a three-level model was developed.

Ethical Considerations

No issues noted.

Clarity of Findings

The results are clearly presented. Prevalence rates and spatial clusters are justified within the results and consistent with extant literature. Key findings and hotspots are adequately documented and discussed. For example, Kapoeta East, Kapoeta South, Kapoeta North, Budi, Pibor, and Ikotos are noted clusters and hotspots. The authors indicate that formal education is protective against overlapping of IPV and one other form of VAWG.

Figures and Tables

While the tables are not ideally formatted for reading/reviewing, they fit within the guidelines of PLOS Global Health and any issues can be dealt with during typesetting. One small issue: the first column should be "Location(s)" in Table 3 as it can possibly refer to multiple locations.

The Figure 1 Spatial Autocorrelation Report does *not* significantly contribute to this manuscript. The only relevant values are reported in the ULH quadrant (Moran's I, z-score, and p-value). Generally, figures should be able to stand alone from the manuscript. The normal distribution plot is well-known and unnecessary for the reader. Moreover, the three inset maps at the bottom of the figure are not specific to the region of interest and merely generic maps and default maps produced by ArcGIS and ESRI software. If this figure is to be included, the authors should find more ways to tie it directly to the findings and justify its ability to stand independent from the manuscript. Otherwise, it should be eliminated. No issues noted with Figure or Figure 3.

Statistical Rigor

Statistics and findings are correctly reported and interpreted. No major issues noted.

In the multilevel model in Table 4, the decrease of 0.05 (5 percentage points) indicates that the added predictors have explained a modest portion of the county-level variance. However, 56% of the variance is still attributable to county-level factors, suggesting that a significant amount of clustering at the county level remains unexplained. Discussion on this finding should be expanded. Sensitivity analyses are appropriate and adequately described in the Supplementary Files.

5. Discussion

Interpretation

- Are the findings interpreted in the context of existing literature?

- Do the authors explain why certain hotspots (e.g., Kapoeta counties) emerged?

Implications

- Are the implications for policy and intervention clear and actionable?

- Does the discussion address the role of education and community literacy in reducing VAWG attitudes?

Limitations

- Are study limitations (e.g., use of older data, county-level focus) sufficiently discussed?

6. Conclusion

The conclusion is succinct and appropriately summarizes and contextualizes the findings.

The authors suggest that the findings advocate for increasing education for children (upstream), and addressing gender inequities in these regions to address the issues of IPV and VAWG. However, an expanded discussion by region and feasibility would be helpful to make the findings more actionable. More Specific recommendations of interventions or programmatic direction would be beneficial to the manuscript.

7. Writing Quality

This manuscript is well-written and has minimal writing errors or issues. The language is accessible and appropriate for the intended audience. The authors use appropriate language and avoid specious and causal language. Units of measurement (i.e., distance) are consistent throughout the manuscript.

8. Suggestions for Improvement

Gender Stratification

- Consider conducting gender-disaggregated analyses of the clusters to determine whether there are significant differences in overlapping attitudes between men and women.
- Incorporate into spatial analyses

Cultural Factors

- Explore cultural or historical factors behind clusters.
- Supports more causal directions

Geographic Resolution

- Improve the resolution of spatial data (e.g., sub-county level).
- Supports the multilevel analyses

Difference-In-Differences or Regression Discontinuity Analyses

- Explore whether exposure to humanitarian crises (such as displacement) or specific interventions in some regions influenced attitudinal shifts

9. Final Assessment

Overall Strengths

The main strength of this analysis is the quality of the dataset used and array of spatial analyses applied to the research questions. The authors should be commended for their careful parsing and analysis of the Survey.

Overall Weaknesses

The main weakness of this study is that more discussion is needed on the limitations of the dataset and the sampling methods (as described above). A minor weakness of this study is the minor flaws in formatting tables and figures.

Recommendation

Given the aforementioned issues, I recommend that this manuscript be accepted with minor revisions.

The authors should be commended for this contribution to PLOS Global Health. I look forward to the revisions.

Kind Regards,

Review 3
