# Supplementary material for: Prevalence, locations and predictors of attitudes accepting both intimate partner violence and additional forms of violence against women and girls in South Sudan: a geospatial analysis
Source: PLOS Glob Public Health. 2025 Apr 9;5(4):e0004144. doi: 10.1371/journal.pgph.0004144 (PMC11981127; doi:10.1371/journal.pgph.0004144)
Supplement: Attachment — Submitted filename: GIS responses v2_18Dec.docx [file pgph.0004144.s006.docx]

18 December 2024

TO: The Editor

All of the co-authors are grateful to the reviewers for their helpful comments.

PGPH-D-24-01742
Prevalence, locations and predictors of attitudes accepting both intimate partner violence and additional forms of violence against women and girls in South Sudan: a geospatial analysis
PLOS Global Public Health.

We structured our responses as follows:

I. Responses to Editor comments
II. Responses to peer reviewer 1
III. Responses to peer reviewer 2
IV. Responses to peer reviewer 3.
I. **RESPONSES TO EDITOR COMMENTS**
   1. **The reviewers and I found the manuscript to be well-written and provide important insights into spatial factors associated with gender-based violence in South Sudan. Given the ongoing humanitarian crises in South Sudan, and the difficulties in conducting research in this setting, the manuscript fills an important gap.**

Thank you so very much.

2. **However, please note areas where the manuscript could be improved. Specifically, the reviewers raised several questions concerning the sampling approach and methodology that need to be clarified. Consider whether all tables and figures are appropriately formatted and integrated with the text.**

Thanks for your comment. We have provided an answer to each question and comment. Also, we edited the tables and figures to meet PLoS Global Public Health’s requirements.

3. **Finally, consider the suggestions regarding the discussion, particularly in terms of whether the literature referenced could be updated, and whether you might be able to better situate the findings of your study in the context of the literature on gender-based and humanitarian crises.**

We have updated the literature referencing in the introduction and discussion sections. However, as our topic is attitudes about VAWG instead of acts of VAWG, the literature available is more limited. As a result, we retained some of the references used in the previous draft. Likewise, we did not eliminate studies such as the WHO multi-country study on women's health and domestic violence against women. Its results (2005) on VAWG prevalence, health outcomes and women's responses because it represents a milestone in gender-based violence research.

Additionally, we edited our discussion in order to present our findings in a context of humanitarian crises.

**RESPONSES TO REVIEWER 1**

**Reviewer #1: Its a suggestion to amend the conclusion by describing only the variable (married women) as the most high likely ratio of associated with IPV. 11 % of the references were old than 10 years. try to replace them with new ones.**

Thanks for your helpful comment. However, to focus exclusively on describing the association of married partners accepting VAWG in the conclusion could distract readers from taking on board the beneficial effect of education on attitudes rejecting VAWG. However, we have attempted to be sensitive to your recommendation when possible.

Additionally, we updated the literature referencing in the introduction and discussion sections when possible. However, as in our earlier response, our topic is attitudes towards VAWG instead of actions of VAWG, the available literature is limited. As a result, we retained some references from the previous draft which are seminal works. For example, we did not eliminate studies such as the WHO multi-country study on women's health and domestic violence against women (2005) which recorded VAWG prevalence, health outcomes and women's responses to VAWG.

**RESPONSES TO REVIEWER 2**

**Reviewer #2: The research needs major modifications and some important components of the research has been missed. So, try to revise the manuscript based on the provided comments. In addtion, follow the Plos Global Public Health journal policies on how to prepare manuscripts.**

**ABSTRACT**

1. **Page 3, line 23-51: Add the sub titles of the abstract such as Introduction, Objective, Methodology, Result, Conclusion and recommendation…**

Thanks for your comment. We added an Introduction, Methodology, Results, and Conclusion subheading. We did not include them initially because the instruction found in [Submission Guidelines | PLOS Global Public Health](https://journals.plos.org/globalpublichealth/s/submission-guidelines#loc-manuscript-organization), indicates that the Abstract should not include headings.

2. **Page 2, line 24-28: Rewrite the sentence… “if attitudes accepting IPV overlap with attitudes accepting additional forms of VAWG …” like “This research aimed to determine the prevalence of attitudes accepting IPV overlap with additional forms of VAWG…”**

Thanks for your comment. We agreed with you and fixed the paragraph accordingly.

3. **Page 2, line 37-38: Rewrite the sentence… The prevalence of attitudes accepting IPV overlapped with attitudes accepting at least one other form of VAWG was 34.72% (95%CI: 33.14-36.34) of the time.**

Thanks for your comment. We fixed the paragraph according to your earlier suggestion.

4. **Page 2, line 41-44: What do you mean OR? Is it Adusted odds ratio or Crude odds ration? Make it clear for the readers. It can be corrected as “People married, cohabiting or living together (AOR(OR=1.40, 95%CI=1.04-1.90) as well as people widowed, divorced or separated (AOR (OR= 1.75, 95%CI=1.05-2.93) were associated with attitudinal overlaps. Conversely, communities with any formal education were associated with a lower odds of overlapping (AOR (OR=0.26. 95%CI=0.09-0.70).”**

Thanks for your comment. We agreed with you and modified our wording from OR to aOR throughout the manuscript.

5. **Page 3, line 48-50: Please write your conclusions and recommendations based on your findings.**

We very much appreciate your comment and rephrased the conclusion in the abstract.

**INTRODUCTION**

6. **Page 4, line 61-66: Why you use all paragraphs as capital letters? Please write your basic reasons unless use the small letters as you used in other paragraphs.**

We corrected this mistake in formatting.

7. **Page 4, line 63-65: Please add other risk factors identified after reviewing different recent literatures.**

We have added additional risk factors such as those from the systematic review of risk factors of VAWG in high-prevalence settings (2022) (10.1136/bmjgh-2021-007704) and the Socioecological framework for drivers of conflicts and postconflict VAWG (2022) (10.1177/10778012221094065).

**MATERIALS AND METHODS**

**Study design and sample size**

8. **Page 6, line 131-132: Of the interviewed participants, how many of them where females and males individually?**

Our study population included 1732 females and 1730 males.

9. **Page 7, line 136-146: The sampling technique is not clearly explained. The way of the determining the sampling size was not also clearly explained. So, please rewrite in detail for understanding for the readers more in figures.**

We edited the text about sampling and sampling size and referred readers to references to familiarize themselves about LQAS principles.

10. **Page 7, line 156: How do you check the consistency of data and languages translations? Explain more within the manuscript.**

We rewrote lines 169-181 (track changes version) according to your suggestion.

Regarding translations we added a sentence on page 8 describing out backtranslation. “Translated instruments were backtranslated into English to ensure that the original meaning of the question was retained.”

Regarding data consistency we added additional information at the bottom of page 8, “Supervisors were responsible for checking inconsistencies (duplications, erroneous entries) or missing data daily. If any of these issues were identified once data were uploaded to our server, the supervisor immediately assisted the interviewer to fix the error and/or obtain the missing data.”

11. **Page 10, line 208: The prevalence of what? Please rewrite the word aligned with your objectives.**

We modified the subheading to “Prevalence of attitudes towards IPV and other forms of VAWG”

12. **Page 10, line 215: The Spatial analysis of what? Please rewrite the word aligned with your objectives.**

We modified the subheading to “Spatial analysis of overlapping attitudes towards IPV and at least one other form of VAWG”.

13. **Page 11, line 237: The Multilevel regression of what? Please rewrite the word aligned with your objectives.**

We appreciate a lot your comment. We modified the subheading to “Multilevel regression of overlapping attitudes towards IPV and at least one other form of VAWG”.

14. **Page 12, line 251-252: When would be the multicollinearity test conducted? Is it during Multilevel regression or before? Write your explanation and corrections in brief.**

We re-structured the paragraph in lines 257-276 (track record version) to display the steps we used to highlight the logical and chronological order.

**RESULTS**

15. **Page 12, line 270: Which sentence is more appropriate? Characteristics of the sample or Characteristics of the participants? Explain your justification.**

In the section to which you refer we want to describe participant characteristics. Therefore, we modified the subheading to “characteristic of the participants”.

16. **Page 13, line 271-272: What is your response rate?**

The response rate was 99.4%

17. **Page 13, line 278-281: Table 1 is not written as standard of writing tables. Please rewrite the table.**

We edited the table according to the PloS Global Public Health formatting guidance.

18. **Page 14, line 282: The prevalence of what? Please rewrite the word with the topic you want to explain.**

Thank for your comment. We modified the subheading to “Prevalence of attitudes towards IPV and other forms of VAWG”.

19. **Page 13, line 290-291: update the table with standard way of table presentation (the comment is for all tables).**

We edited the table according to the PloS Global Public Health formatting guidance.

20. **Page 16, line 308-326: There is the figure 1, 2 and 3? Please add the figures within your manuscript.**

While we thank you for your comment the instruction found in [Figures | PLOS Global Public Health](https://journals.plos.org/globalpublichealth/s/figures), instructs authors to include the caption only in the manuscript. Figures should be included in a separate file.

21. **Page 18, line 341-342: Please clarify OR whether Adjusted or crude odds ratio.**

All our models were adjusted either using individual or county/regional data or both. We have modified our wording from OR to aOR throughout the manuscript.

22. **Page 18-20, line 346-349: Please add the p-value of individual independent variables (Both Bilevel and Multilevel logistic regression) with in the table for clear understanding of the association for general readers.**

We included p-values in table 4 as you advised.

23. **Page 21, line 359-363: Please clarify OR whether Adjusted or crude odds ratio? The comment is applicable throughout the manuscript.**

We modified our wording from OR to aOR throughout the manuscript.

24. **Page 22, line 397-401: I am not clear about your explanation here. It that result or discussion component of the manuscript? Please discuss your finding in detail as compare to other research findings both for higher and level prevalence of overlapping acceptance of IPV and at least one other form of VAWG and lastly suggest your reasons for it.**

We edited lines 430-440 (track changes version) according to your suggestions.

25. **Page 22, line 399: Only use one style of reference either Vancouver or Harvard style. Don’t use both within single research.**

We edited the sentences using the Vancouver reference style.

26. **Page 24, line 426-432: Show the findings in table or add the table number which shows this finding for general readers.**

We added the table number showing the finding in result section.

27. **Page 25, line 467: Use the most recent world bank report if available.**

We would be happy to update this point. However, World Bank does not have more recent data about literacy level in South Sudan. Nevertheless, we have updated our references.

28. **Page 25, line 474-478: Make sure that you discussed are align with your findings. Don’t add or explain any other factors which are not related with your findings.**

We deleted the paragraph mentioned in order to keep us into our findings.

29. **Page 26, line 493-498: This is not a strength of the study rather it is the recommendation. Please use this at a recommendation part of your research. Note that, your recommendation should be based on your findings.**

We relocated the lines mentioned above to “Conclusion and recommendations”.

30. **Page 28, line 527-228: please add the title of recommendation here or together as conclusion like “Conclusions and recommendations” on line 510. As general comments, some components of the manuscripts are messing such as 1. Ethical considerations 2. Abbreviations/Acronyms 3. Availability of data and materials 4. Funding 5. Computing interest 6. Authors contributions 7. Authors information if needed**

Thanks for your comments. We edited the text accordingly to your suggestions as follows:

a) We replaced the subheading “Conclusion” for “Conclusions and recommendations”.
b) Regarding ethical considerations, they are included in Materials and Methods.
c) We did not include a list with abbreviations/Acronymus because according to the information provided by PloS Global Public Health ([Submission Guidelines | PLOS Global Public Health](https://journals.plos.org/globalpublichealth/s/submission-guidelines)), these should be define upon first appearance in the text. However, if it is needed, the list is:

VAWG	:	Violence against women and girls

IPV	:	Intimate partner violence

OR	:	Odds ratio

WHO	:	World health organization

AA	:	Administrative area

NHS	:	National household survey

LQAS	:	Lot quality assurance sampling

SA	:	Supervision area

PPS	:	Probability proportional to size

UNICEF	:	United Nations Children´s Fund

LSTM	:	Liverpool school of tropical medicine

ODK	:	Open data kit

LLR	:	Likelihood ratio test

VIF	:	Variance inflation factor

SD	:	Standard deviation

CI	:	Confidence interval

aOR	:	Adjusted odds ratio

ICC	:	Intraclass correlation coefficient

d) Availability of data and materials were included in the online submission system where we stated the following:

“data is available from UNICEF South Sudan which can be contacted through <https://www.unicef.org/southsudan/contact>”

e) Funding: As in our earlier comment, our financial disclosure was stated in the submission as “Funding for the South Sudan National Household Survey was provided through a grant from UNICEF South Sudan (SSD/PCA2019614). The funders had no role in the study design, data collection and analysis, decision to publish, or preparation of the manuscript. The research reported in this paper received no specific grant from any funding agency in the public, commercial or not-for-profit sectors.”
f) Competing interests: As before, our competing interests were state in the submission to PLoS GPH as they instructed: “The authors have declared that no competing interests exist.”
g) Author contribution: Thanks for your comments. We fixed this missing information reporting the author contribution as follows:

“AL, ILE, JJV, RL conceptualised the study. CJ had oversight of the statistical analyses. RA, RL and JJV collected the data. AL and ILE implemented the preliminary analysis and literature search and contributed equally to this study. AL, ILE, CJ and JJV implemented the final analysis. All authors drafted and reviewed the manuscript. AL and ILE contributed equally to this study.”

h) Author information if needed: We added “corresponding author” together with the email of Professor Joseph Valadez on page 1 line 21.

**RESPONSES TO REVIEWER 3**

1. **GENERAL OVERVIEW**

**In this original research article, the authors present novel analyses of IPV and VAWG in South Sudan. Using the National Household Survey South Sudan 2020, and a suite of spatial analyses the authors conclude that acceptance of IPV and at least one other form of VAGW is spatially clustered. The authors provide foundational policy recommendations and insights that can inform women’s empowerment and support international policy initiatives.**

Thank you.

2. **INTRODUCTION**

**The authors properly situate Violence Against Women and Girls as a significant global public health issue and human rights violation. Causal investigations of VAGW focus on gender dynamics and patriarchal normativity. Considering these issues, the authors indicate three aims: (i) calculate attitudinal prevalence of acceptance of IPW and overlapping attitudes toward IPW and one form of VAGW, (ii) analyze clusters, and (iii) identify relevant explanatory factors for these attitudes.**

Thank you.

3. **METHODS**

**3.1 Study Design**

**This study is a cross-sectional study on a secondary analysis of data from the National Household Survey of South Sudan, 2020. This dataset is appropriate for the research aims and goals. Outcomes are clearly defined. Geographical groupings are logical and supported by literature.**

Thank you.

**3.2 Sampling and Data Collection**

**LQAS is somewhat insufficiently explained as a sampling method. A brief passage on its justification would support the readership’s comprehension. For example, “It is commonly applied in public health and manufacturing to assess whether certain quality or performance thresholds are met without requiring a full population survey.”**

**The use of PPS is appropriate for this analysis. Importantly, PPS requires accurate population estimates to determine sampling probabilities. If the population data is outdated, incomplete, or inaccurate (authors indicated using 2008 census for a conflict-affected country like South Sudan), the sample may not represent the current population distribution. In regions with high migration, displacement, or population changes, PPS might oversample or under sample certain areas. PPS tends to under sample sparsely populated areas, which may lead to different results. Despite this, these methodological concerns should be discussed more completely.**

Thank you for your comment. We rewrote the text about sampling and sampling size and refer readers to our references to familiarize themselves with LQAS method. We also edited lines 567-581 to clarify the limitations of working with estimations based on the Census of 2008.

**3.3 Spatial Analysis**

**The use Local Indicators of Spatial Association (LISAs) is a appropriate class of spatial analysis for addressing the aims of the present study. Global Moran’s I is an appropriate measure of clustering. Getis and Ord's local Gi* are appropriate measures for Hotspot Analysis. No issues are noted here.**

Thank you.

**3.4 Multilevel Analysis**

**Multilevel models are appropriately described and statistically sound. The authors indicate a three-level model was developed.**

Thank you.

**3.5 Ethical Considerations**

**No issues noted.**

Thank you.

**3.6 Clarity of Findings**

**The results are clearly presented. Prevalence rates and spatial clusters are justified within the results and consistent with extant literature. Key findings and hotspots are adequately documented and discussed. For example, Kapoeta East, Kapoeta South, Kapoeta North, Budi, Pibor, and Ikotos are noted clusters and hotspots. The authors indicate that formal education is protective against overlapping of IPV and one other form of VAWG.**

Thank you.

**3.7 Figures and Tables**

**While the tables are not ideally formatted for reading/reviewing, they fit within the guidelines of PLOS Global Health and any issues can be dealt with during typesetting. One small issue: the first column should be “Location(s)” in Table 3 as it can possibly refer to multiple locations. The Figure 1 Spatial Autocorrelation Report does not significantly contribute to this manuscript. The only relevant values are reported in the ULH quadrant (Moran’s I, z-score, and p-value). Generally, figures should be able to stand alone from the manuscript. The normal distribution plot is well-known and unnecessary for the reader. Moreover, the three inset maps at the bottom of the figure are not specific to the region of interest and merely generic maps and default maps produced by ArcGIS and ESRI software. If this figure is to be included, the authors should find more ways to tie it directly to the findings and justify its ability to stand independent from the manuscript. Otherwise, it should be eliminated. No issues noted with Figure or Figure 3.**

We edited the table according to the PloS Global Public Health formatting guidance.

In Table 3, we changed the word “Location” by “Location(s)”.

Regarding Figure 1, we agreed that its inclusion in the manuscript is not crucial because the results are explained in the text. However, we also agree with previous research [1-4] and consider it a basic step in reporting geospatial analysis. As a result, we located the Figure as a supplementary file.

**3.8 Statistical Rigor**

**Statistics and findings are correctly reported and interpreted. No major issues noted. In the multilevel model in Table 4, the decrease of 0.05 (5 percentage points) indicates that the added predictors have explained a modest portion of the county-level variance. However, 56% of the variance is still attributable to county-level factors, suggesting that a significant amount of clustering at the county level remains unexplained. Discussion on this finding should be expanded. Sensitivity analyses are appropriate and adequately described in the Supplementary Files.**

We also note 56% of the variance in the overlap in attitudes accepting IPV and other expression of VAWG is due to variation between clusters.

While 44% of the variation is unexplained by the model, we also state that 44% of the variation is attributed to differences among participants within counties. Accordingly, we edited lines 484-488 (track changes version). In the conclusion and recommendations (lines 613-620, track changes version) we also modified the text.

4. **DISCUSSION**

**4.1 Interpretation**

- **Are the findings interpreted in the context of existing literature?**

We do think our findings are interpreted according to the existing literature. We included previous research conducted in South Sudan as well as in other low-income-countries and humanitarian settings to inform our findings.

- **Do the authors explain why certain hotspots (e.g., Kapoeta counties) emerged?**

In order to deepen our explanation about the hotspots and cluster identified, we edited lines 450-464 (track changes version) according to your suggestions.

**4.2 Implications**

- **Are the implications for policy and intervention clear and actionable?**

Yes, in lines 610-631 (track changes version) we recommend clear and actionable interventions to address VAWG in South Sudan.

- **Does the discussion address the role of education and community literacy in reducing VAWG attitudes?**

In order to be more precise about the discussion on benefit of education at the community level, we edited lines 524-539 (track changes version). Likewise, we re-structured the wording to improve the readability.

**4.3 Limitations**

- **Are study limitations (e.g., use of older data, county-level focus) sufficiently discussed?**

In order to expand our discussion about the limitation due to the use of the 2008 Census projection to estimate our sample frame we edited lines 574-584 (track changes version).

Likewise, regarding data analysed at the county level, we edited lines 585-590 (track changes version).

5. **CONCLUSION**

**The conclusion is succinct and appropriately summarizes and contextualizes the findings. The authors suggest that the findings advocate for increasing education for children (upstream), and addressing gender inequities in these regions to address the issues of IPV and VAWG. However, an expanded discussion by region and feasibility would be helpful to make the findings more actionable. More Specific recommendations of interventions or programmatic direction would be beneficial to the manuscript.**

While we agree that more specific recommendations for interventions or programmatic directions would be beneficial to the manuscript, we think that what is first needed is an in-depth analysis of the South Sudanese situation to suggest concrete improvements by region. This is due to the humanitarian condition of the country and the unstable political and economic situation. We also recommend that qualitative data are needed to guide the design of implementation research.

6. **WRITING QUALITY**

**This manuscript is well-written and has minimal writing errors or issues. The language is accessible and appropriate for the intended audience. The authors use appropriate language and avoid specious and causal language. Units of measurement (i.e., distance) are consistent throughout the manuscript.**

Thanks for your comment.

7. **SUGGESTIONS FOR IMPROVEMENT**
   7.1 **Gender Stratification**

- **Consider conducting gender-disaggregated analyses of the clusters to determine whether there are significant differences in overlapping attitudes between men and women.**

Thanks for your comment. We should note that it was not the aim of our research to conduct an analysis by gender. However, the number of participants per county disaggregated by gender would reduce considerably the sample sizes which could impact on the robustness of our findings in the spatial analysis.

We would be happy to explore, in future research, attitudes towards VAWG by gender.

- **Incorporate into spatial analyses**

See our earlier explanation.

7.2 **Cultural Factors**
- **Explore cultural or historical factors behind clusters.**

The historical context within the clusters is majorly the same as the rest of South Sudan, except Pibor in which the practice of raiding of women and children during cattle rustling is a norm. We have included this context in our revised discussion. The key distinguishing factor between Kapoeta East, Kapoeta South, and Kapoeta North from the surrounding counties is the tribe of the main inhabitants, which is associated with a nomadic-pastoral way of life and cattle raiding. Although we have not established a direct link between the culture of the Toposa tribe and VAWG, we have contextualized it within their nomadic pastoralist way of life, which is associated with cattle rustling. These contextual differences have been added to the discussion.

- **Supports more causal directions**

Causality is very difficult to ascertain given the cultural basis of the VAWG. To ascertain possible causality linkages an intervention study could be designed. However, we think we are not justified to comment on this topic just now.

7.3 **Geographic Resolution**
- **Improve the resolution of spatial data (e.g., sub-county level).**

Thanks for your comment. However, providing additional lower level administrative boundaries to the maps (payams) may not be needed because we analysed and displayed the results at county level. Nevertheless, the maps display the higher administrative state boundaries.

- **Supports the multilevel analyses**

We conducted a multilevel analysis in a spatial analysis as spatial analysis often exhibits hierarchical structure and dependence that need explicit modelling. In this regard, multilevel analysis allows us to account for the variability across individuals and counties and evaluate the association of outcome and risk factors.

7.4 **Difference-In-Differences or Regression Discontinuity Analyses**
- **Explore whether exposure to humanitarian crises (such as displacement) or specific interventions in some regions influenced attitudinal shifts**

Our analysis plan was based on previous research in Sub-Saharan Africa analysing VAWG through spatial analysis [1, 2, 4, 5] which used multilevel regression analyses which is appropriate to account for variability across multiple levels. Difference-in-differences or regression discontinuity analyses are beyond our current research intentions but could be considered at a later date as a separate paper**.**

Similarly, we included variables that were included in the National Household Survey 2020. Unfortunately, displacement or additional variables were not available for consideration.

8. **FINAL ASSESSMENT**

**8.1 Overall Strengths**

**The main strength of this analysis is the quality of the dataset used and array of spatial analyses applied to the research questions. The authors should be commended for their careful parsing and analysis of the Survey.**

Thanks for your comment.

8.2 **Overall Weaknesses**

**The main weakness of this study is that more discussion is needed on the limitations of the dataset and the sampling methods (as described above). A minor weakness of this study is the minor flaws in formatting tables and figures.**

Thank you. As you see above, we have edited the discussion, specifically providing more details about the limitations. Furthermore, we have edited the tables in figures according to PloS instructions.

**8.3Recommendation**

**Given the aforementioned issues, I recommend that this manuscript be accepted with minor revisions. The authors should be commended for this contribution to PLOS Global Health. I look forward to the revisions.**

Thanks for your comment.

**Kind Regards, Review 3**

1.	Angaw DA, Melesse AW, Geremew BM, Tesema GA. Spatial distribution and determinants of intimate partner violence among reproductive-age women in Ethiopia: Spatial and Multilevel analysis. BMC Women's Health. 2021;21(1):1-13. doi: 10.1186/s12905-021-01218-3.

2.	Bolarinwa OA, Tessema ZT, Okyere J, Ahinkorah BO, Seidu A-A. Spatial distribution and predictors of lifetime experience of intimate partner violence among women in South Africa. PLOS Glob Public Health. 2023;3(1):e0000920. doi: 10.1371/journal.pgph.0000920.

3.	Tusa BS, Kebede SA, Weldesenbet AB. Spatial distribution and determinant factors of intimate partner violence among reproductive age group women in Ethiopia: Using generalized structural equation modeling. PLoS One. 2022;17(2):e0263811. doi: 10.1371/journal.pone.0263811.

4.	Bolarinwa OA, Ahinkorah BO, Frimpong JB, Seidu A-A, Tessema ZT. Spatial distribution and predictors of intimate partner violence among women in Nigeria. BMC Women's Health. 2022;22(1):1-245. doi: 10.1186/s12905-022-01823-w.

5.	Seid E, Melese T, Alemu K. Spatial distribution and predictors of domestic violence against women: evidence from analysis of Ethiopian demographic health survey 2016. BMC Women's Health. 2021;21(1):1-15. doi: 10.1186/s12905-021-01465-4.
